# A Study of the Strength Performance of Peat Soil: A Modified Cement-Based Stabilization Agent Using Fly Ash and Polypropylene Fiber

**DOI:** 10.3390/polym13234059

**Published:** 2021-11-23

**Authors:** Mohammed K. H. Radwan, Foo Wei Lee, Yoke Bee Woon, Ming Kun Yew, Kim Hung Mo, Soon Han Wai

**Affiliations:** 1 Department of Civil Engineering, Lee Kong Chian Faculty of Engineering and Science, Universiti Tunku Abdul Rahman, Kajang 43000, Malaysia; eng.moh.rad@1utar.my (M.K.H.R.); woonyb@utar.edu.my (Y.B.W.); yewmk@utar.edu.my (M.K.Y.); 2 Department of Civil Engineering, Faculty of Engineering, Universiti Malaya, Kuala Lumpur 50603, Malaysia; khmo@um.edu.my; 3 Department of Environmental Engineering, Faculty of Engineering and Green Technology, Universiti Tunku Abdul Rahman, Kampar 31900, Malaysia; waish@utar.edu.my

**Keywords:** peat soil, cement, stabilization, fly ash, polypropylene fiber, unconfined compressive strength (UCS), California bearing ratio (CBR), scanning electron microscopy (SEM)

## Abstract

The use of cement as a soil stabilization agent is one of the common solutions to enhancing the engineering properties of soil. However, the impact and cost of using cement have raised environmental concerns, generating much interest in the search for alternative materials to reduce the use of cement as a stabilizing agent in soil treatment. This study looked into limiting cement content in peat soil stabilization by using fly ash waste and polypropylene fiber (PPF). It focused on soil mechanical mediation for stabilization of peat with fly ash cement and PPF cement by comparing the mechanical properties, using unconfined compressive strength (UCS) and California bearing ratio (CBR) tests. The control (untreated) peat specimen and specimens with either fly ash (10%, 20% and 30%) and PPF (0.1%, 0.15% and 0.2%) were studied. Test results showed that 30% of fly ash and cement content displays the highest UCS and CBR values and gives the most reliable compressibility properties. On the other hand, UCS and CBR test results indicate optimum values of PPF–cement stabilizing agent content in the specimen of 0.15% PPF and 30% cement. Selected specimens were analyzed using scanning electron microscopy (SEM), and PPF threads were found to be well surrounded by cement-stabilized peat matrices. It was also observed that the specimen with 30% fly ash generated more hydration products when compared to the specimen with 100% cement content. It is concluded that the use of fly ash cement and PPF cement as stabilizing agents to limit the cement usage in peat soil treatment is potentially viable.

## 1. Introduction

Around 8% of the total area of the world’s land is covered by peat soil, and Malaysia is ranked ninth by total peat area with approximately 25,000 km^2^ [1]. Peat deposits are formed from the accumulation and fossilization of partly decomposed and fragmented remains of plants [2]. It is relatively easy to differentiate peat from other soil types by its dark brown to black coloration, high organic content, high moisture content and lightweight nature [3].

Peat is categorized as a type of soft soil and is inappropriate for geotechnical applications due to its extreme compressibility (high porosity), high squeezability and low shear strength [4]. In addition, peat soil is often referred to as problematic soil due to its high acidity, high rates of creep, high water content (500–2000%) and high permeability [5]. These properties could cause differential settlement or failures in structures built on such soils, making peat soil an unsuitable material for construction.

There have been numerous studies on the feasibility of using cement as a stabilizing agent to enhance the strength properties of peat soil [6,7,8]. These studies have found that cement-based stabilizing agents generally improve the compressive strength of treated peat soil. It has also been proven that cement stabilization exhibits superior performance in enhancing the soil properties of peat soils with various characteristics and at different geographical locations [9]. The hydration reactions of cement and the formation of hydration products, such as calcium silicate (C–S–H) and calcium aluminate (C–A–H) hydrates, due to cement-based soil treatment, enhance the strength development of soil [10]. For example, calcium hydroxide (Ca(OH)_2_), a cement hydration product, may react with silica or alumina sources in soil, contributing to enhancing the long-term strength of the treated soil [11]. However, cement production is known to emit a considerable amount of carbon dioxide (CO_2_) into the atmosphere [12].

In order to reduce its impact on the environment, several types of industrial waste have been introduced to fully or partially replace cement as a soil stabilizer [13]. For example, Said and Taib [14] studied the use of carbide lime in peat soil stabilization. They found that the unconfined compressive strength (UCS) improved when increasing amounts of carbide lime were added to peat soil. On the other hand, Kolay and Pui [15] utilized fly ash and gypsum to stabilize peat soil, and reported that the use of fly ash gave better UCS results than gypsum. Pond ash [6], sodium bentonite [16] and clayey diatomite [5] were also used to stabilize peat soil. As efforts to search for other suitable waste materials to replace cement in soil treatment continued, it was found that soil treated with cement-waste-based stabilizer agents exhibits low flexural and tensile strength, and in some cases, even brittle behaviour [16,17]. However, very few studies have discussed and compared the use of recycled waste such as fly ash and polypropylene fiber (PPF) as peat stabilization agents in peat soil treatment.

Therefore, this study focused on investigating the mechanical properties of peat stabilized with different percentages of cement content (i.e., 10%, 20% and 30%), as the conventional peat soil stabilizer. The strength performance of cement-treated peat soil with added fly ash (10%, 20% and 30%) or PPF (0.1%, 0.15% and 0.2%) was also examined. UCS and CBR tests were conducted to evaluate the strength performance of each soil stabilizing material.

## 2. Materials and Methods

### 2.1. Peat Sample

**Figure 1:**
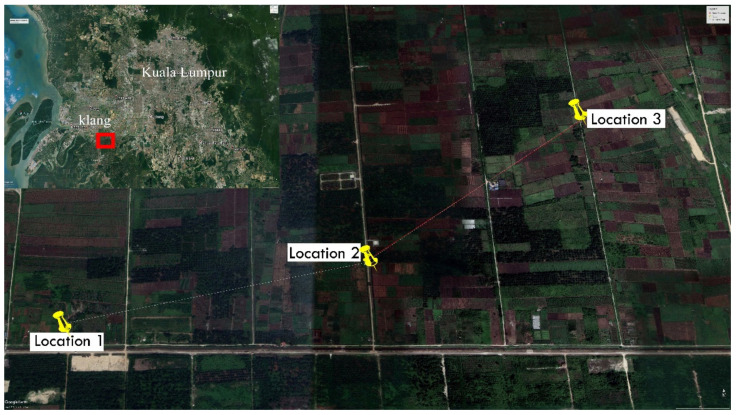
Locations of peat soil sampling in this study.

Peat soil samples in this study were acquired from three different locations approximately 1 km apart in Johan Setia village, Klang city, located about 40 km away from Kuala Lumpur, as shown in Figure 1. The primary geotechnical properties of in-situ (untreated) peat soil are displayed in Table 1. The soil samples were obtained at depths between 0.5 and 1 m below the existing ground surface. It should be noted that the groundwater level was found to be about 0.4 m below the existing ground surface. This accounts for the high moisture content found in the peat soil samples. The physical appearance of peat soil revealed that it was fully saturated and had very dark brown coloration. Peat soil sampler was implemented in this study to determine the physical and chemical properties of peat soil. The sampler was used in a previous study by Zulkifley et al. [18]. The sampler mainly consists of a movable chamber that prevents the occurrence of disturbances, an anchored fin and a movable sampling chamber. Samples were collected using hand augers and were then oven-dried at 60 °C until a constant weight was observed (average duration of 48 h). The oven-dried samples were then stored in plastic bags after they reached room temperature.

### 2.2. Characteristics of Cement and Fly Ash

**Figure 2:**
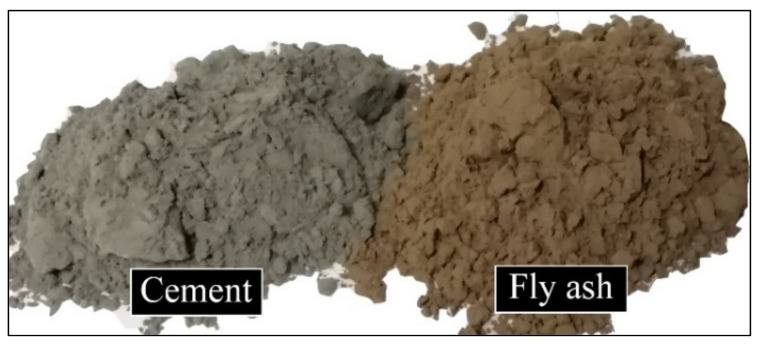
Physical appearances of cement and fly ash (left to right).

Ordinary Portland cement (OPC) was used in this study—cement type CEM I (Grade 42.5N/52.5N) with a specific gravity of 3.15, complying with MS EN 197-1. Coal fly ash with a specific gravity of 2.9 was used to reduce cement content, and it is classified as Class “C” in accordance with ASTM C618-17a [19]. The physical appearances of cement and fly ash are presented in Figure 2, and Table 2 lists the chemical composition, loss of ignition (LOI) and specific surface area of each of these materials.

### 2.3. Characteristics of PPF

**Figure 3:**
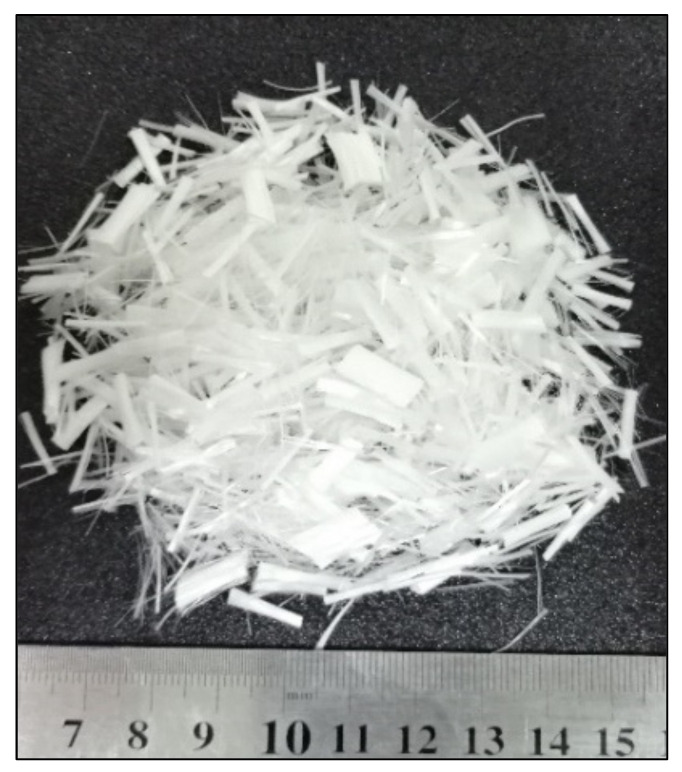
The physical appearance of PPF.

PPFs of 12 mm in length to aid the flexural behaviour of treated peat soil were used in this study (see Figure 3). The physical properties of PPF, as provided by the manufacturer, are listed in Table 3.

### 2.4. Stabilizer Combinations and Sample Preparation

The proposed stabilizer combinations and proportions in each peat sample are listed in Table 4. In Phase 1, an untreated peat control specimen (labelled as U0) was prepared first. This was followed by the preparation of C10, C20 and C30 samples, where 10%, 20% and 30% cement by weight, respectively, was added to untreated peat. The cement contents could be described in weight per unit volume as 130, 140, and 150 kg/m^3^. Thereafter, a series of tests were conducted. In Phase 2, the cement-treated peat was modified by adding fly ash (10%, 20% and 30% by weight, equal to 128, 137, and 146 kg/m^3^, respectively). Besides that, three different levels of PPF (0.1% (1.2 kg/m^3^), 0.15% (1.8 kg/m^3^) and 0.2% (2.4 kg/m^3^) by volume) were added to the peat samples, and similar tests were conducted on those samples.

The treated peat soil samples were each hand mixed in a mixing bowl for about 5–10 min until the mixture was homogeneous. The treated peat was then poured into 76 cm high cylindrical moulds of 38 mm in diameter. The moulds were demoulded the following day and placed in a water tank to be cured under laboratory conditions at 30 ± 2 °C until the desired testing days (days 7, 14 and 28).

## 3. Test Programme

### 3.1. pH Value Test

The pH values of untreated and treated peat soil were measured using a digital pH meter in accordance with ASTM D2976 (2015). First, 30 g of dry peat soil sample was sieved through a 200 μm sieve and mixed with distilled water. The sample was then stirred for 10 min to attain a homogenous mixture. The samples were left to stand overnight before pH measurements were taken.

### 3.2. Proctor Compaction Test

The Proctor compaction test was conducted to obtain the maximum dry density (MDD) and the optimum moisture content (OMC) values of untreated and treated peat soils in accordance with ASTM D 698 (2007). Before testing, the collected soil samples were oven-dried at 110 °C for four hours. They were then sieved using sieve no.4 (4.75 mm) and stored in sealed plastic storage boxes for further testing. Initially, 8% of water was assumed (per weight of dry peat soil), and the water content was then increased by 2% for each trial. Three layers of soil were then fed to the pre-weighted mould. Compaction was carried out using a 4.5 kg rammer by striking 27 blows with a uniform rate at about 1.5 s per drop. Each layer of compaction was about one-third of the volume of the mould. Next, the surface of the soil was trimmed off using a straight edge. The weight of the mould, together with the compacted soil, was recorded. The soil sample was then demoulded and oven-dried for 24 h. After 24 h, the sample was kept at room temperature to cool down and the dried mass was then recorded. This process was repeated five times for each sample’s moisture content and the average density was recorded.

### 3.3. Unconfined Compressive Strength (UCS) Test

Upon the determination of OMC for each soil stabilizer, three specimens of treated and untreated peat soil were prepared according to ASTM D 2166 (2016) for UCS testing. The peat specimen was first mixed with its corresponding OMC. The mixed soil was then poured into cylindrical moulds (76 mm heigh by 38 mm diameter) and compacted into three layers. After 24 h, the specimens were extracted vertically using a hydraulic jack and cured in water before 7-day, 14-day and 28-day tests were conducted on the samples for UCS. The rate of strain was fixed at 1 mm/min.

### 3.4. California Bearing Ratio (CBR) Test

CBR tests to evaluate the strengths of the proposed soil stabilizers were conducted in accordance with ASTM D1883. The contents of cement, fly ash and PPF were mixed manually with the peat soil until reaching homogeneity in their OMC. The samples were then tested for UCS. The CBR moulds containing the soil samples were soaked in water to simulate actual in-situ conditions until the day of testing.

### 3.5. Morphology Analysis

Twenty-eight-day stabilized soil specimens were examined using a scanning electron microscope (SEM, Phenom XL Desktop, Thermo Fisher Scientific, Waltham, MA, USA). After the UCS test, small slices were collected from the cores of the samples. The sliced samples were dried using an electric laboratory oven at 60 °C for four hours to remove moisture and air vacuumed to remove dust particles. A Phenom ProX desktop microscope (Phenom XL Desktop, Thermo Fisher Scientific, MA, USA) was used to observe the morphologies of the stabilized soil samples. Scanning was conducted at an acceleration voltage of 2.0 kV and with a magnification of 2000×. An attached X-ray energy dispersive spectroscope (EDS, OmniProbe 200, Oxford Instruments, Abingdon, United Kingdom) was used to characterize and identify the elementary compositions of the soils.

## 4. Results and Discussions

### 4.1. Proctor Compaction Test

**Figure 4:**
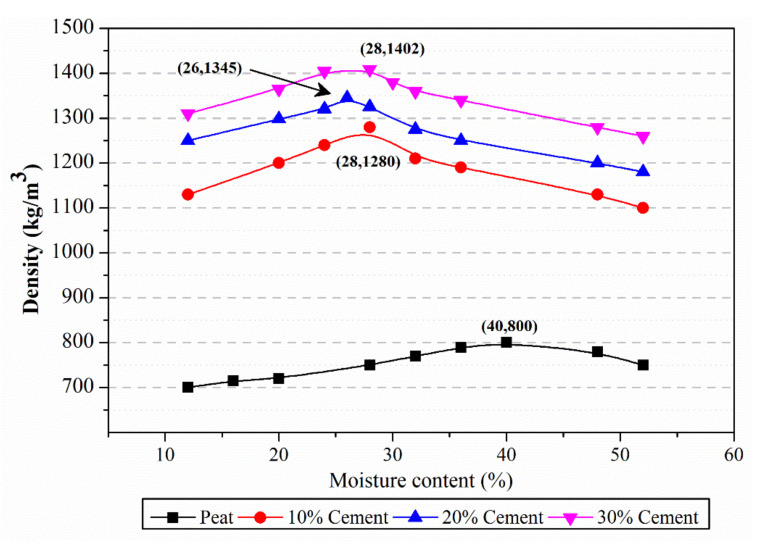
Optimum moisture content—maximum dry density diagram.

The results of OMC for the corresponding MDD for both the untreated and cement-treated peat soil are depicted in Figure 4. The results show that the untreated peat soil attained an MDD of 800 kg/m^3^ when 40% moisture was utilized. One possible reason for such a high OMC is due to the presence of large voids, as organic contents and water are absorbed to fill up the large voids. This is in line with Zulkifley et al.’s description of peat soil having rather low bulk density and high water content due to the presence of high organic contents, such as branches, trunks, roots, leaves and fruits. This is also substantiated by literature showing a similar bulk density range of 800–1000 kg/m^3^ in West Malaysian peat [20].

Values of MDD and OMC were found to be 1250 kg/m^3^ and 32%, respectively, for stabilized peat soil with 10% OPC. MDD values for treated peat soil specimens were found to increase when OPC content was increased from 10% to 30%. This behaviour was a direct result of increasing the solid-to-organic content ratio and the higher specific gravity of peat soil with cement. On the other hand, OMC values were found to decrease when OPC content was increased. The low optimum moisture contents in these specimens were probably due to the voids being occupied by the presence of cement. Regardless of the amounts of cement, samples of treated peat soil showed consistent trends of increasing MDD and reducing OMC. This was due to the addition of cement, which in turn introduced further solid (non-organic) content to the peat soil. These observations are in line with the results obtained from Boobathiraja et al. [8].

### 4.2. pH Test

**Figure 5:**
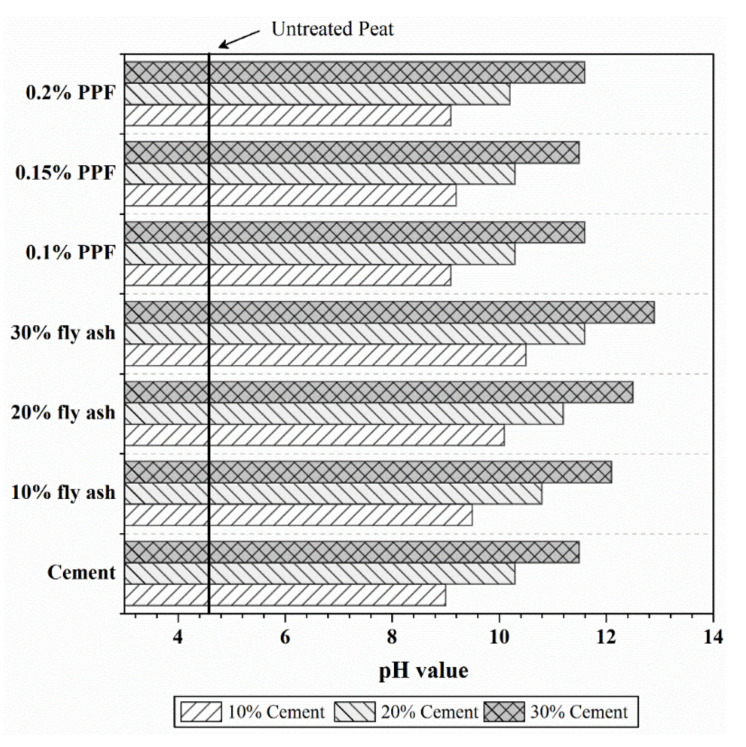
pH values of treated and untreated peat soil.

pH tests were conducted to determine the pH values of treated and untreated peat soil. The results are presented in Figure 5. The average pH value of untreated peat soil was found to be 4.5, indicating the acidic nature of peat soil. This value corresponds to the previously measured pH value of around 3.76 for local peat soil obtained from different Malaysian states [21]. The low pH value of peat soil is due to the presence of high amounts of decomposed plant fragments (organic), which influence the chemical and biological properties of peat soil [22].

The addition of cement turns the peat into an alkaline material. This was demonstrated by an increase in pH value to 11.5 when 10–30% of cement was added to peat soil. This correlates with the findings by Ghadir and Ranjbar [23], who reported the treated soil’s pH values to be between 11 and 12 when cement dosages of 5% to 15% were added. A study by Moayedi and Nazir [22] also revealed that the release of OH- in remarkable amounts resulted in pH values of between 10 and 12 in stabilized peat soil.

The pH values of fly ash-treated soil were also found to increase with the addition of fly ash. Furthermore, pH increments of approximately 0.5 units per 10% increase in fly ash dosage were observed. This finding is consistent with the report from Nath et al. [24]. On the other hand, no significant changes were observed in the pH values of PPF-treated soil specimens. The slight changes in pH values in these specimens were likely due to the presence of cement in peat soil specimens.

### 4.3. Unconfined Compressive Strength Test

**Figure 6:**
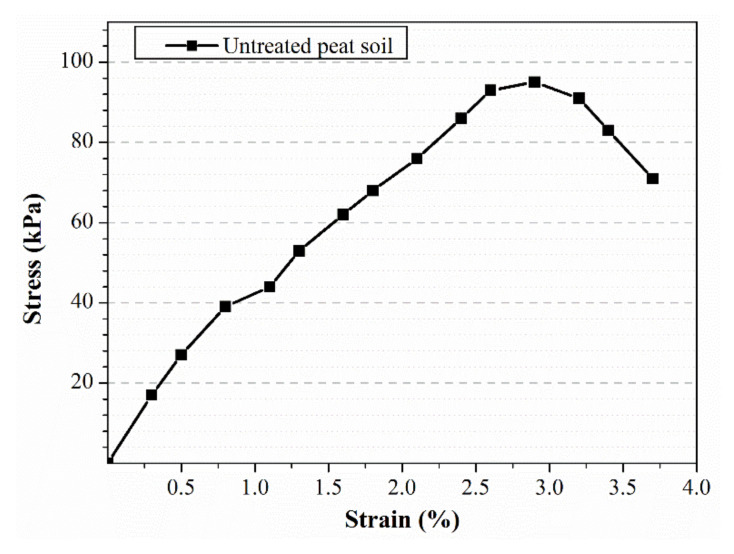
UCS of untreated peat soil.

Figure 6 shows the stress–strain diagram of untreated peat soil. The untreated peat soil had a maximum UCS of 95 kPa, corresponding to approximately 2.9% strain. This value lies within the range of reported strengths in previous research [7]. Compared to other soils (such as sand [25], clay [26,27], Laterite, Kaolin [21], etc.), peat soil exhibits lower UCS. This is mainly due to the presence of large amounts of water content, voids and organic content in peat soil [21].

#### 4.3.1. Effect of Cement Addition on UCS

**Figure 7:**
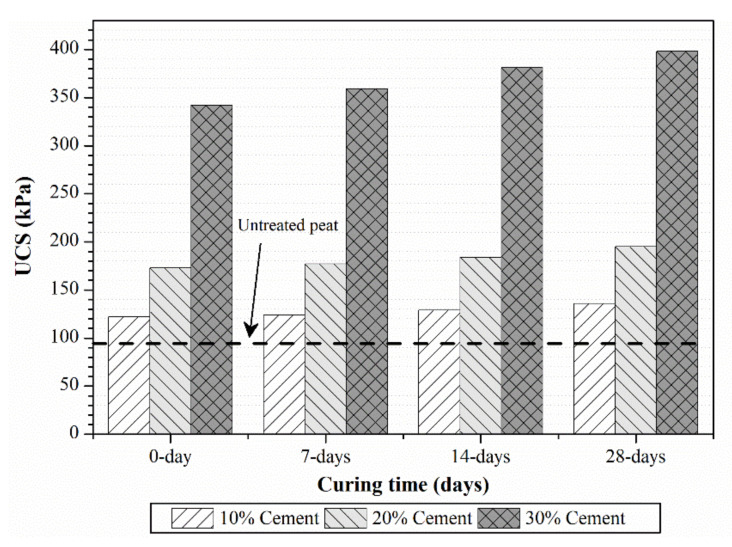
UCS of peat soil treated with different mix proportions of cement.

UCS test results for each proportion of cement content are presented in Figure 7. The UCS of cement-treated peat soil was found to increase with the curing period. This could be attributed to the additional hydration reactions that occur during curing [28], which in turn would have enhanced the strength characteristics of cement-treated peat soil. It was also observed that at all ages, higher UCS was attained by cement-treated peat soil as the cement content in the specimens was increased. For instance, the addition of 20% of cement resulted in about 30% higher UCS when compared to the 10% addition of cement for 7-day, 14-day and 28-day specimens. Likewise, when the cement dosage was increased up to 30%, the UCS increased by around 50% when compared to the UCS of the specimen treated with 20% cement. A similar observation was reported by Kalantari et al. [29].

Table 5 summarizes the percentage improvements in the 28-day-curing test results of UCS for modified cement stabilizer with either fly ash or PPF. The results were calculated based on the differences between modified peat soil and untreated peat soil.

#### 4.3.2. Effect of Fly Ash Addition on UCS

**Figure 8:**
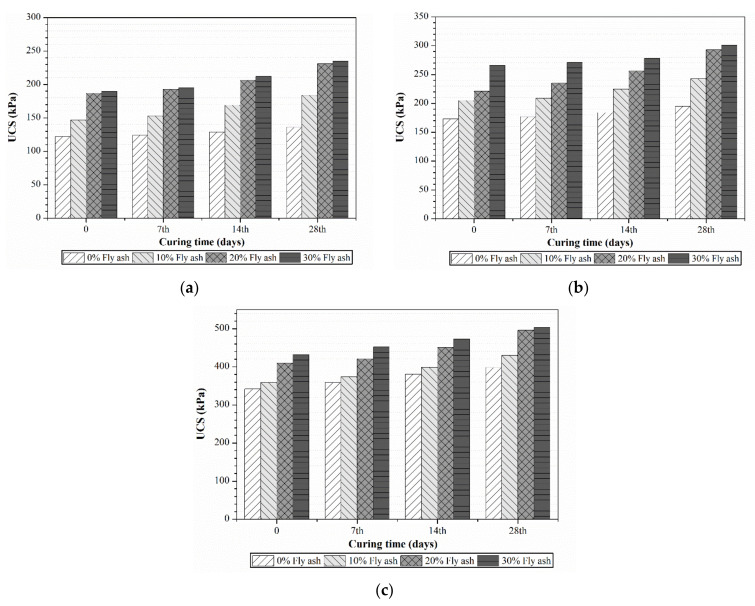
UCS of fly ash–cement-treated peat with (**a**) 10% cement and 0–30% fly ash, (**b**) 20% cement and 0–30% fly ash and (**c**) 30% cement and 0–30% fly ash and different curing durations.

Figure 8 shows UCS results for each combination of fly ash and cement. As fly ash is a cementitious material, fly ash-based test specimens also exhibited similar trends as those reported for solely cement-treated soil test specimens in Section 4.3.1. The addition of 30% fly ash resulted in higher UCS values in all mixtures considered. As expected, fly ash–cement combination specimens outperformed cement-treated specimens in terms of UCS (see Table 5). This might have been due to the hydration products produced during pozzolanic reactions that take place in the presence of fly ash. The release of calcium hydroxide (Ca(OH)_2_), a by-product in the hydration process, activates the pozzolanic activity of fly ash, resulting in a gain in strength. The higher amount of fly ash is attributed to the increase in the filler effect of fly ash and the additional pozzolanic reaction products from fly ash.

#### 4.3.3. Effect of PPF Addition on Unconfined Compressive Strength

**Figure 9:**
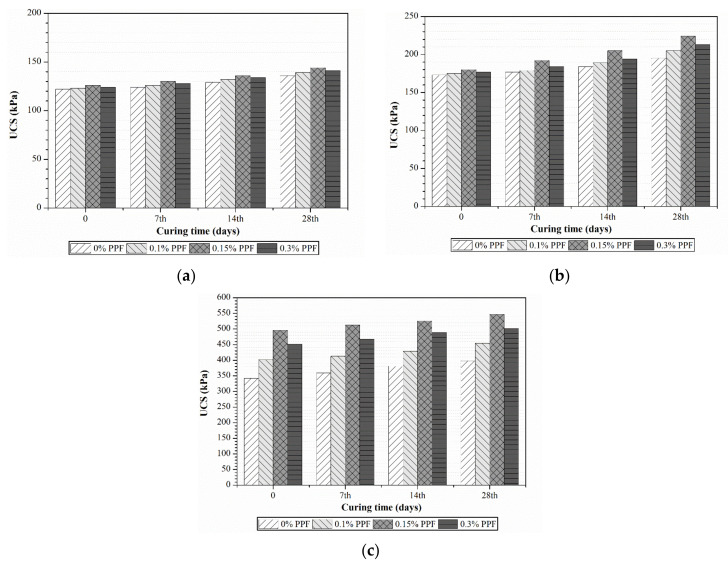
UCS of treated peat with (**a**) 10% cement and 0–0.2% PPF, (**b**) 20% cement and 0–0.2% PPF and (**c**) 30% cement and 0–0.2% PPF and different curing durations.

Figure 9a–c presents the UCS test results for 7, 14 and 28 day cures obtained from different proportions of PPF (0.1–0.2%) added to cement-treated peat soil. The addition of PPF was shown to increase the UCS of treated peat soil, particularly in samples with the 0.15% PPF and 30% cement combination. The comparison of UCS between PPF–cement samples and untreated peat soil samples is listed in Table 5. The addition of 0.1% of PPF to 10% and 20% cement-treated peat soils increased the UCS by 2.5 and 5 kPa on average, respectively. However, the UCS increased by around 55% when the same amount of PPF was added to 30% cement-treated peat soil. This might have been due to PPF being chemically inert and due to its ability to bind the cement during the hydration process. Hence, a more remarkable improvement was achieved.

On the other hand, the addition of 0.1% or 0.15% PPF also increased the UCS of cement-treated peat soil. This improvement was mostly due to the load-deformation behaviour through surface friction and interlocking between soil particles, the cement mixture and the PPF [30]. Soil reinforced with PPF exhibits greater toughness and ductility, increased formability and bending strength, and reduced post-peak strength loss compared to cemented-treated soil [31]. This implies that an appropriate amount of polypropylene should be considered as a stabilizing agent. The drop in UCS values of specimens with more than 2.0% of PPF content could have been due to the uneven distribution and lumping effect of PPF fragments.

### 4.4. California Bearing Ratio Test

**Figure 10:**
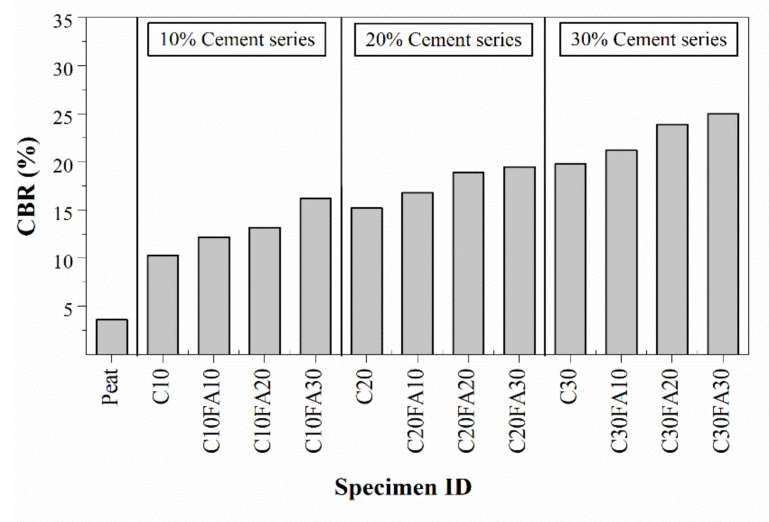
CBR results of fly ash cement-treated peat soil.

**Figure 11:**
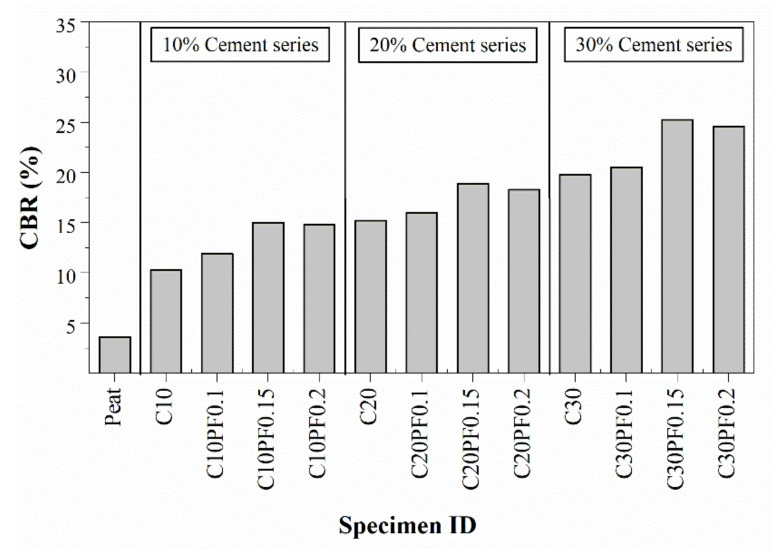
CBR results of PPF-cement-treated peat soil.

The measured CBR test results for different proportions of fly ash cement and PPF-cement-treated peat soil are presented in Figure 10 and Figure 11, respectively. The lowest CBR result observed was around 4% for untreated peat soil. Mohd Yusoff et al. [21] reported a CBR value of around 6.5% for unsoaked peat soil and assigned such a low value to the relative dry weight of peat soil. Thereafter, the addition of cement resulted in a substantial improvement in the CBR performance of peat soil. The results demonstrated that CBR values increased from 3.6% for untreated (U0) peat soil to around 10.3%, 15.2% and 19.8% in the cases of C10, C20 and C30 cement-treated peat soil specimens, respectively. In addition, the inclusion of both fly ash and PPF further enhanced the CBR performance of treated peat soil.

A positive correlation was observed between the CBR value and the amount of fly ash added to soil with all cement percentages, as can be seen in Figure 10. On average, the addition of 10% and 20% of fly ash to cement-treated peat soils gave about 12% and 24% higher CBR values when compared to the corresponding cement-treated peat soils (for all cement series). As an example, C20FA10 treated peat soil exhibited a CBR of 16.8%, whereas C20 (containing only 20% cement) produced a CBR of 15.2%. The largest improvement in CBR values (37% on average) was observed when 30% of fly ash was added to cement-treated peat soil. These observations are in line with UCS results, as the strength of treated peat soil samples improved with cement and fly ash contents.

Similarly, an increase in PPF content produced higher CBR results when compared to the corresponding cement-treated peat soil. Figure 11 shows that there were significant improvements in CBR values when 0.1% and 0.15% of PPF were added to cement-treated peat soils. However, slight reduced CBR values were observed when the amount of PPF was increased up to 0.2%. In all specimens, CBR values of cement-PPF treated peat soil exceeded those of untreated peat soil.

### 4.5. SEM Analysis

**Figure 12:**
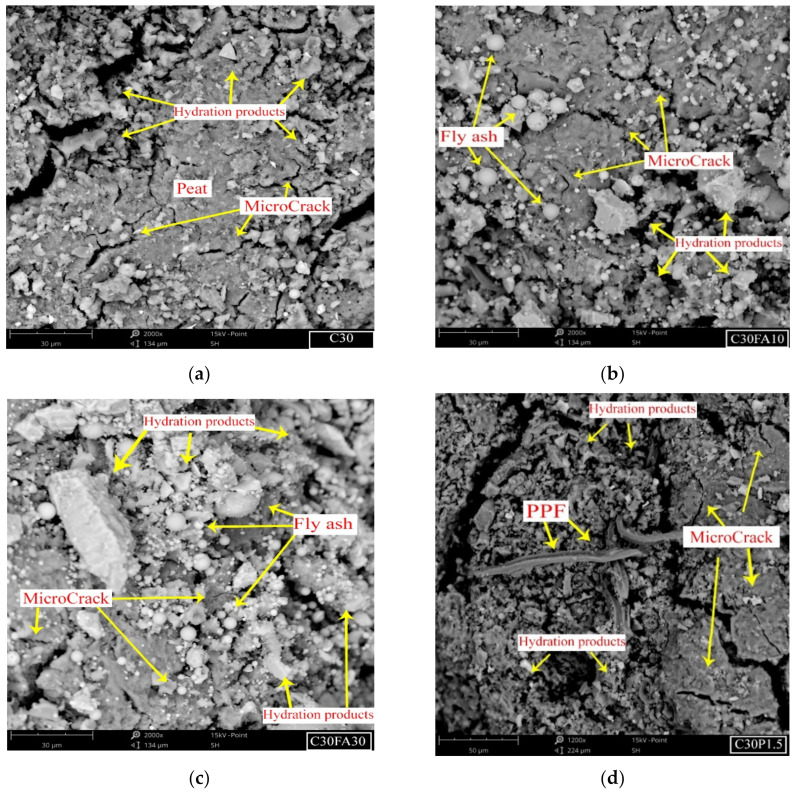
SEM micrographs of stabilized soil after 28 days with (**a**) C30 (30% cement), (**b**) C30FA10, (**c**) 30FA30 and (**d**) C30PF1.5%.

The results of SEM of selected stabilized soil specimens, including C30, C30FA10, C30FA30 and C30PF0.15, are presented in Figure 12. In general, all stabilized soil specimens showed a considerable number of internal microcracks accompanied by hydration products. Figure 12a shows the presence of a small, uneven distribution of microcracks in the stabilized soil with the addition of 30% cement. These microcracks could be attributed to the presence of cement particles, as exhibited by small expansion during the hydration process while the peat soil shrinks as the moisture is reduced. Hydration products are visibly shown to attach to peat particles. Figure 12b,c presents the SEM results of soil stabilized with 30% cement along with 10% or 20% fly ash, respectively. The morphologies of both soil samples demonstrate additional hydration products with fewer microcracks. The additional hydration products could be attributed to fly ash’s presence, which provides additional C–S–H gel throughout the pozzolanic reactions. Unreacted fly ash particles were found to be present in both soil specimens, although it was more pronounced in the C30FA30 sample. This could be attributed to fly ash’s slow pozzolanic reaction rate, which starts upon the formation of calcium hydroxide (CH) from cement hydration [28,32]. The presence of unreacted fly ash also contributed to enhancing the strength through the filler effect. This explained the enhanced UCS results when higher contents of fly ash were added to the soil. Figure 12d shows the morphology of CA30PF1.5 stabilized soil. As expected, significant amounts of hydration products were found to be distributed around the peat particles. It can be seen that the fiber threads were wrapped tightly by the cement hydration products–peat soil matrix, thereby effectively restricting the mobility of the fibre threads. The reduction in movement thus increased the reinforcement benefit of cement-stabilized soil.

**Figure 13:**
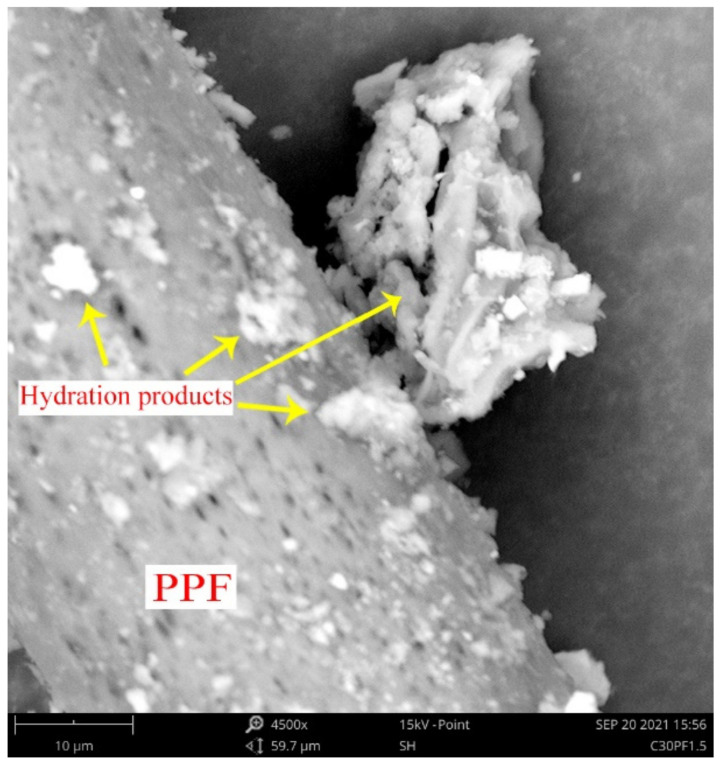
SEM image of C30PF1.5 with a magnification of 4500×.

Figure 13 shows the clear morphology of PPF fiber thread present in the C30PF0.15 specimen under a magnification of 4500×. This clearly demonstrates the ability of the surfaces of PPF threads to bond with cement hydration products, thereby increasing the static friction between fiber and cement–soil matrix, eventually translating to an increase in the strength characteristics of fiber-added, cement-stabilized peat soil [33].

## 5. Conclusions and Recommendations

This study evaluated the use of fly ash (10% to 30%) and polypropylene fiber (PPF) (0.1% to 0.2%) as additives to enhance cement-stabilized peat soil. MDD, OMC, pH, unconfined compressive strength (UCS) and California bearing ratio (CBR) tests were conducted on these specimens. The morphologies of stabilized soil were also examined using SEM–EDS analysis. Based on the results, the following conclusions can be drawn:

- The utilization of cement as a conventional soil stabilizing agent substantially enhanced the basic properties (pH, MDD and OMC) of peat soil, reflecting the improved geotechnical performances of treated peat soil. Higher cement content resulted in superior improvements in the UCS and CBR of cement-treated peat soil.
- Further enhancements in UCS and CBR values were attained by adding 10%, 20% and 30% of fly ash in cement-treated peat soil. The use of 30% of fly ash in the 30% cement-treated peat soil resulted in a 430% improvement in UCS and a 595% improvement in CBR.
- The addition of 0.15% PPF was found to be the optimum dosage of PPF, exhibiting a 476% improvement in UCS and a 600% improvement in CBR.
- SEM analysis depicted the presence of cement hydration products attached to the peat soil particles. The number of hydration products increased with the addition of fly ash. However, unreacted fly ash particles were presented in fly ash cement-stabilized peat soil, which enhanced UCS and CBR performances through the filler effect. A clear cement hydration product appeared on the surfaces of fiber threads—which was effectively surrounded by the cement–peat soil matrix—in PPF-cement-stabilized peat soil (i.e., C30PF1.5).

Based on the enhanced geotechnical properties obtained from this study, the use of both fly ash and polypropylene fiber can potentially limit the usage of cement in future peat soil treatments. The addition of 30% of fly ash and 0.15% of polypropylene fiber showed potential improvements in soil properties, even when no additional cement was added. For future research, it is recommended that other geotechnical soil properties, such as consistency limits, swelling parameters and undrained shear strength be further investigated. In addition, the influence of changes in flexural strength and stiffness could be considered in future investigations on the effects of PPF on cement-treated soil. The combined effects of using fly ash and PPF as cement-based stabilizer agents should also be investigated to determine the significant effects on the binding process of hydration products. It would also be interesting to study the crystalline phases presented in treated soil using X-ray diffraction analysis using different dosages of stabilizer and additives.

## Figures and Tables

**Table 1:** Physical properties of untreated peat soil.

Property	Standard and Specifications	Value
Depth of sampling (m)	-	0.5–1
Natural moisture content (%)	ASTM D2216	598.5
Fiber content (%)	ASTM D1997	79.33
Loss on Ignition (%)	ASTM D2974	90.84
Organic content (%)	ASTM D2974	90.47
Liquid limit (%)	BS EN 1997-2: 2006	200.2
Plastic limit (%)		Non plastic
Specific gravity (Gs)	AASHTO T 180-D	1.21
pH	BS EN 1997-2: 2006	4.5

**Table 2:** Chemical composition and specific surfaces of the binders used.

Binder	Composition (%)							LOI	Specific Surface Area (cm^2^/g)
	Na_2_O	Al_2_O_3_	SiO_2_	MgO	CaO	Fe_2_O_3_	SO_3_		
OPC	0.1	4.5	18.7	3.3	64.8	4.1	3	3.2	3110
Fly ash	3.1	16.1	35.1	8.1	15.3	17.3	2.4	2.5	3800

**Table 3:** Physical properties of PPF.

Properties	Length (mm)	Diameter (µm)	Density (g/cm^3^)	Tensile Strength (MPa)	Modulus of Elasticity (GPa)	Elongation at Break (%)
PPF	12	50	0.9	350–500	3.5–3.9	12.9

**Table 4:** Mix designs of the stabilized peat specimens.

	Specimen ID	Material Content (%)		
		Cement	Fly Ash	PPF
Phase 1	U0	0	0	0
	C10	10	0	0
	C20	20	0	0
	C30	30	0	0
Phase 2	C10FA	10	(10%, 20% and 30%)	0
	C20FA	20	(10%, 20% and 30%)	0
	C30FA	30	(10%, 20% and 30%)	0
	C10PF	10	0	(0.1%, 0.15% and 0.2%)
	C20PF	20	0	(0.1%, 0.15% and 0.2%)
	C30PF	30	0	(0.1%, 0.15% and 0.2%)

**Table 5:** Summary of UCS improvements (%) with added fly ash and PPF.

Untreated Peat	Cement		Fly Ash + Cement		PPF + Cement	
	Cement Content	UCS Improvement (%)	Fly Ash Content	UCS Improvement (%)	PPF Content	UCS Improvement (%)
95 KPa	10%	43.2	10%	93.7	0.10%	46.3
			20%	143.2	0.15%	51.6
			30%	147.4	0.20%	48.4
	20%	105.3	10%	155.8	0.10%	115.8
			20%	208.4	0.15%	135.8
			30%	216.8	0.20%	124.2
	30%	319	10%	352.6	0.10%	377.9
			20%	422.1	**0.15%**	**475.8**
			**30%**	**429.5**	0.20%	427.4
